# Associations of Internalizing and Externalizing Problems in Childhood and Adolescence With Adult Labor Market Marginalization

**DOI:** 10.1001/jamanetworkopen.2023.17905

**Published:** 2023-06-08

**Authors:** Iman Alaie, Pia Svedberg, Annina Ropponen, Jurgita Narusyte

**Affiliations:** 1 Division of Insurance Medicine, Department of Clinical Neuroscience, Karolinska Institutet, Stockholm, Sweden; 2 Division of Child and Adolescent Psychiatry, Department of Medical Sciences, Uppsala University, Uppsala, Sweden; 3 Finnish Institute of Occupational Health, Helsinki, Finland

## Abstract

**Importance:**

Mental health problems in early life are associated with labor market marginalization, especially in youths with persistent internalizing and externalizing problems. However, previous research has not adjusted for familial (genetic and shared environmental) factors.

**Objective:**

To examine associations of early-life internalizing and externalizing problems with adulthood unemployment and work disability, adjusting for familial factors.

**Design, Setting, and Participants:**

This population-based prospective cohort study included Swedish twins who were born in 1985-1986 and surveyed at 4 consecutive waves across childhood and adolescence until 2005. Through linkage to nationwide registries, participants were followed up from 2006 to 2018. Data analyses were conducted between September 2022 and April 2023.

**Exposures:**

Internalizing and externalizing problems, assessed with the Child Behavior Checklist. Participants were differentiated regarding duration of internalizing and externalizing problems (persistent, episodic, and noncases).

**Main Outcomes and Measures:**

Unemployment (180 days or more of being unemployed) and work disability (60 days or more of being sickness absent or disability pensioned) during follow-up. Cox proportional hazards regression models were calculated to obtain cause-specific hazard ratios (HRs) with 95% CIs in the whole cohort and exposure-discordant twin pairs.

**Results:**

Of 2845 participants, 1464 (51.5%) were female. Incident unemployment was experienced by 944 (33.2%) and incident work disability by 522 (18.3%) participants. Compared with noncases, persistent internalizing problems were associated with unemployment (HR, 1.56; 95% CI, 1.27-1.92) and work disability (HR, 2.32; 95% CI, 1.80-2.99). Similarly, compared with noncases, persistent externalizing problems were associated with unemployment (HR, 1.87; 95% CI, 1.55-2.26) and work disability (HR, 2.38; 95% CI, 1.87-3.03). Persistent cases had overall higher risks of adverse outcomes than episodic cases. After adjustment for familial factors, associations with unemployment were no longer statistically significant, whereas associations with work disability remained or were only slightly reduced.

**Conclusions and Relevance:**

In this cohort study of young Swedish twins, familial factors explained the associations between early-life persistent internalizing and externalizing problems and unemployment; such factors were comparatively less important for the association with work disability. This suggests nonshared environmental factors may be important for the risk of future work disability among young individuals with persistent internalizing and externalizing problems.

## Key Points

**Question:**

Are early-life internalizing and externalizing problems associated with adulthood unemployment and work disability, and do familial factors (genetics and shared environment) play a role in the association?

**Findings:**

In this cohort study of 2845 young Swedish twins prospectively followed over the transition to adulthood, both persistent internalizing and externalizing problems were associated with unemployment and work disability. After adjustment for familial factors, there was no association with unemployment, but associations with work disability remained similar.

**Meaning:**

These findings suggest that nonshared environmental factors may play a role in the longitudinal association of early-life mental health problems with adult work disability.

## Teaser

This cohort study of 2845 Swedish twins and their parents examines the association of mental health problems experienced during childhood and adolescence and unemployment later in life.

## Introduction

Mental health disorders are among the leading causes of the burden of disease in youths and adults worldwide.^1,2^ There is growing recognition that internalizing (eg, depression, anxiety) and externalizing (eg, disruptive conduct, substance use) disorders are key contributors to labor market marginalization (LMM) today,^3,4^ as a substantial proportion of those affected are less likely to be employed and, if employed, more likely to be on sickness absence or disability pension (often termed “work disability”).^5,6^ Cross-national comparative data imply that up to 50% of all public expenditures on work disability benefits are due to mental health disorders; among young adults, this figure moves toward 50% to 80% across several Western societies.^5,6,7^ Furthermore, in Sweden, the labor market situation for young adults has been particularly problematic over the past decade, with unemployment rates higher than in many other societies.^8^ Concerns have therefore been raised that long-term receipt of social benefits may trap economically inactive young adults in poverty and welfare dependency over the longer term.^5,6^ This speaks to the importance of advancing empirical evidence in this area to help reduce the individual and societal burden.

Through a preventive and economic lens, this bleak picture raises questions about the key determinants of LMM among young individuals affected by mental health problems. This is especially important given that many internalizing and externalizing disorders are common already in childhood and adolescence,^9,10^ and even more common when including subclinical psychopathology below the established diagnostic thresholds.^11^ Several longitudinal studies have demonstrated associations between youth mental health problems and broad harms to overall life prospects, including social, economic, and health-related adversities.^12,13,14,15^ However, a critical limitation of most previous studies on affected youths is the lack of control for unmeasured familial factors (ie, genetics and early-life family environment),^16^ which emerges as particularly relevant insofar as genetic vulnerability is known to contribute to the risk of both developing mental health problems^17^ and experiencing work disability.^18,19,20,21,22,23,24^

Twin studies have shown that genetics accounts for 42% to 49% of the variance in disability pension due to mental disorders,^18,19^ and 33% to 49% of the variance in all-cause sickness absence.^20,21,22^ Furthermore, a longitudinal twin study on young adult employees showed that the association between lifetime internalizing disorders and sickness absence granted for somatic diseases was influenced by genetic factors alone, while the association with sickness absence granted for mental disorders was influenced by both genetic and nonshared environmental factors.^24^ These studies collectively suggest that the largest share of individual differences in work disability seems to be explained by nonshared environmental factors unique to the individual, that is, experiences not shared by siblings within a twin pair. Conversely, with 1 exception,^18^ these studies found no evidence of effects from shared environmental factors, including the early-life family environment shared within a twin pair. Together, these studies highlight the importance of accounting for potential confounding by familial factors when estimating the risk for unemployment and work disability.

While previous genetically informative studies reporting on the association of youth mental health problems with adulthood LMM suggest that unmeasured familial confounding is highly relevant,^23,25,26^ important knowledge gaps remain to be addressed. First, in terms of duration, there is a need to tease apart the course of mental health problems across childhood and adolescence in relation to LMM, notably as studies suggest persistent cases fare worse than episodic cases.^27,28,29,30,31,32^ This poorer long-term prognosis for persistent cases is observed for both internalizing^27,28,29^ and externalizing problems,^30,31^ and applies to those assessed with structured clinical interviews or self-report questionnaires. Next, in terms of timing, it still remains unclear if the onset of mental health problems in childhood or adolescence is more harmful to adult health and functioning, as current evidence is limited and inconsistent.^23,25,28,29^ Some research suggests adolescent-onset depression is more likely to be associated with long-term negative outcomes than childhood-onset depression,^28^ while other research finds no difference when looking at internalizing and externalizing problems more broadly.^29^

We aimed to investigate associations of early-life internalizing and externalizing problems with adult unemployment and work disability using a Swedish population-based cohort of twins prospectively followed from childhood to adulthood. Our objectives were (1) to examine whether common childhood mental health problems are associated with elevated risk for LMM when taking aspects of the duration and timing of such problems into account, and (2) to assess potential confounding by familial factors.

## Methods

### Study Design and Population

Data were drawn from the Swedish Twin Project of Disability Pension and Sickness Absence (STODS).^33^ Participants were originally included in a population-based longitudinal twin study, the Twin Study of Child and Adolescent Development (TCHAD),^34^ comprising all twins born in Sweden between May 1985 and December 1986 (2960 children in total). In TCHAD, the twins and/or their parents were surveyed on 4 waves of measurement, when the twins were aged 8 to 9 years (wave 1), 13 to 14 years (wave 2), 16 to 17 years (wave 3), and 19 to 20 years (wave 4). At each wave, a comprehensive battery of questionnaires on physical and mental health was mailed to all participants. Response rates were 75%, 73%, 74%, and 78% for parents (waves 1 to 4), and 78%, 82%, and 59% for twin individuals (waves 2 to 4).

In STODS, extensive pseudonymized data from several nationwide registries were retrieved to obtain information on a range of indicators on health and social functioning (eg, sickness absence benefits, unemployment benefits). The personal identification number, which is assigned to all Swedish residents,^35^ enabled linkage of the previously collected survey data with consecutive annual registry-based data. After completion of wave 4, in 2005, twins were followed over a 13-year period until the first incidences of 2 distinct outcomes (ie, long-term unemployment, work disability), emigration or death (ie, censoring reasons), or the end of follow-up (ie, 2018), whichever came first. In the retained cohort (2845 individuals), there were 1064 monozygotic (MZ), 764 dizygotic (DZ) same-sexed, and 844 DZ opposite-sexed twin individuals, and 173 with unknown or missing information.

This study was approved by the Regional Ethical Review Board in Stockholm. Informed consent was obtained in writing from all TCHAD participants, who were also informed about registry-based linkages. This report follows the Strengthening the Reporting of Observational Studies in Epidemiology (STROBE) reporting guideline.

### Exposures

The Child Behavior Checklist (CBCL),^36^ a widely used instrument for assessment of child adaptive behaviors and problem behaviors, was used for exposure classification. The CBCL is based on a Likert-scale response format and comprises items that load onto 2 broad-band scales: internalizing problems and externalizing problems. The CBCL for ages 6 to 18 years was completed by parents at waves 1 through 4, and the CBCL/Youth Self-Report was completed by twins at waves 2 through 4. Caseness at a particular wave was counted as present if reported by either parent, twin individual, or both, and defined as T-scores 65 or above on the relevant scale (eg, internalizing problems) according to recommended cut-off scores.^36,37^

To differentiate the twin individuals regarding duration and timing of childhood or adolescent mental health problems, we applied the following criteria. For duration of internalizing and externalizing problems, we classified twin individuals who met the cut-off at 2 or more waves as persistent cases, and those who met the cut-off at only 1 wave as episodic cases. Those who did not meet the cut-off at any wave were identified as noncases. For those with internalizing or externalizing problems or both (eg, first externalizing then internalizing, or vice versa), persistent cases were defined by meeting the cut-off at 2 or more waves, while episodic cases were defined by meeting the cut-off for internalizing or externalizing problems at only 1 wave. Noncases were those who did not meet the cut-off at any wave. Regarding timing, cases with early-onset were identified as those meeting the cut-off at ages 8 to 9 or 13 to 14 years, whereas cases with later-onset were identified as those meeting the cut-off at ages 16 to 17 or 19 to 20 years.

### Outcomes

Consistent with prior research,^27,38,39^ we defined adult LMM as 2 outcomes, long-term unemployment and work disability, during the follow-up from 2006 to 2018. Long-term unemployment (recorded as a binary outcome) was operationalized as 180 or more annual net days of being registered as full-time or part-time unemployed or included in labor market policy programs. In Sweden, an individual needs to be unemployed, able to work, and ready to accept work that is offered in order to qualify for unemployment benefits. Also, the beneficiary must have worked for at least 6 months prior to becoming unemployed. Employment-related data were retrieved from the Longitudinal Integration Database for Health Insurance and Labor Market Studies (LISA),^40^ administrated by Statistics Sweden.

Work disability (recorded as a binary outcome) was operationalized as 60 or more annual net days of being registered as sickness absent or being granted disability pension. In Sweden, all residents aged 16 to 65 years who have an income from work, unemployment, or other social benefits are eligible for sickness absence benefits if there is a reduced work capacity due to disease or injury. For the initial 14 days of a sick-leave spell, an employee receives sick pay from the employer, although there is 1 qualifying day (more for those who are self-employed) without any pay. If work capacity is still reduced after 14 days, the employee can apply for sickness absence benefits. Furthermore, individuals aged 16 to 64 years who have a permanently impaired work capacity are entitled to disability pension. Since 2003, only temporary activity benefits can be granted for young individuals aged 19 to 29 years, whereas those aged 30 to 64 years can be entitled to permanent sickness compensation. Work disability–related data were retrieved from the Micro Data for the Analysis of Social Insurance (MiDAS) database, held by Swedish Social Insurance Agency.^40,41^

### Covariates

Sex (male vs female) and educational level (low or medium with less than 14 combined years vs high with 14 or more years), were included as covariates. Education was categorized according to the highest level achieved across the follow-up based on data from LISA.^40^

### Statistical Analysis

Data analyses were performed from September 2022 to April 2023. We ran Cox proportional hazards regression models to estimate cause-specific hazard ratios (HRs) with 95% CIs for each of the 2 outcomes. Analyses were based on all available data. First, we analyzed all twins as unrelated individuals while correcting standard errors for twin pair clustering. Next, to examine the extent to which the associations between childhood and adolescent mental health problems and LMM were consistent with a causal hypothesis, we conducted a co-twin control analysis^42,43^ including MZ and same-sexed DZ twin pairs. A co-twin control design compares the outcomes in twins who differ from each other on their exposure to a factor of interest. Twins share not only genetics (100% for MZ and, on average, 50% for DZ) but also early-life family environment. By comparing differentially exposed (ie, exposure-discordant) twins within a pair it is possible to adjust for all unmeasured factors shared by the twins. We analyzed exposure-discordant MZ and DZ twin pairs together. We included sociodemographic factors as covariates in further analyses. Also, we ran sensitivity analyses with exposures entered as continuous variables (ie, T-scores averaged across waves of measurement).

Data management and analysis were performed using SAS version 9.4 (SAS Institute Inc) and R version 4.1.3 (R Foundation for Statistical Computing). Statistical significance was set at *P* < .05, and all tests were 2-tailed.

## Results

Of the 2845 participants included in analysis, 1464 (51.5%) were female, with 1297 (45.6%) attaining higher education across both sexes (Table 1). Most twins had no childhood or adolescent internalizing (2084 [73.3%]), externalizing (1999 [70.3%]), or internalizing and/or externalizing (1685 [59.2%]) problems. Among those meeting the cutoff for any internalizing or externalizing problems, there were more episodic (391 [13.7%] for internalizing; 465 [16.3%] for externalizing; 584 [20.5%] for internalizing and/or externalizing) than persistent (248 [8.7%] for internalizing; 259 [9.1%] for externalizing; 454 [16.0%] for internalizing and/or externalizing) cases. First incidences of long-term unemployment and work disability were experienced by 33% and 18%, respectively.

**Table 1.** Descriptive Characteristics of the Retained Cohort

Characteristics	Problems, No. (%) (N = 2845)^a^									
	All	Internalizing			Externalizing			Internalizing/externalizing		
		None	Episodic	Persistent	None	Episodic	Persistent	None	Episodic	Persistent
**Demographics**										
Sex										
Men	1381 (48.5)	1116 (53.6)	142 (36.3)	55 (22.2)	964 (48.2)	227 (48.8)	122 (47.1)	876 (52.0)	272 (46.6)	165 (36.3)
Women	1464 (51.5)	968 (46.4)	249 (63.7)	193 (77.8)	1035 (51.8)	238 (51.2)	137 (52.9)	809 (48.0)	312 (53.4)	289 (63.7)
Zygosity										
Monozygotic	1064 (37.4)	814 (39.1)	140 (35.8)	86 (34.7)	789 (39.5)	174 (37.4)	77 (29.7)	670 (39.8)	227 (38.9)	143 (31.5)
Dizygotic same-sex	764 (26.9)	558 (26.8)	121 (30.9)	82 (33.1)	562 (28.1)	115 (24.7)	84 (32.4)	460 (27.3)	152 (26.0)	149 (32.8)
Dizygotic opposite-sex	844 (29.7)	618 (29.7)	115 (29.4)	73 (29.4)	568 (28.4)	152 (32.7)	86 (33.2)	483 (28.7)	179 (30.7)	144 (31.7)
Unknown or missing	173 (6.1)	94 (4.5)	15 (3.8)	7 (2.8)	80 (4.0)	24 (5.2)	12 (4.6)	72 (4.3)	26 (4.5)	18 (4.0)
Educational level^b^										
Low or medium (<14 y)	1515 (53.3)	1104 (53.0)	204 (52.2)	130 (52.4)	979 (49.0)	278 (59.8)	181 (69.9)	835 (49.6)	327 (56.0)	276 (60.8)
High (≥14 y)	1297 (45.6)	956 (45.9)	181 (46.3)	117 (47.2)	995 (49.8)	181 (38.9)	78 (30.1)	828 (49.1)	249 (42.6)	177 (39.0)
**Adult labor market marginalization outcomes**										
Long-term unemployment^c^										
Yes	944 (33.2)	639 (30.7)	156 (39.9)	110 (44.4)	604 (30.2)	172 (37.0)	129 (49.8)	492 (29.2)	205 (35.1)	208 (45.8)
No	1901 (66.8)	1445 (69.3)	235 (60.1)	138 (55.6)	1395 (69.8)	293 (63.0)	130 (50.2)	1193 (70.8)	379 (64.9)	246 (54.2)
Follow-up time, mean (SD), y	9.34 (4.99)	9.59 (4.91)	8.63 (5.12)	8.37 (5.22)	9.64 (4.88)	8.98 (5.11)	7.67 (5.26)	9.74 (4.84)	9.11 (5.05)	8.13 (5.25)
Work disability^c^										
Yes	522 (18.3)	321 (15.4)	92 (23.5)	79 (31.9)	309 (15.5)	97 (20.9)	86 (33.2)	239 (14.2)	113 (19.3)	140 (30.8)
No	2323 (81.7)	1763 (84.6)	299 (76.5)	169 (68.1)	1690 (84.5)	368 (79.1)	173 (66.8)	1446 (85.8)	471 (80.7)	314 (69.2)
Follow-up time, mean (SD), y	11.40 (3.37)	11.70 (3.13)	10.90 (3.77)	10.40 (4.12)	11.60 (3.25)	11.40 (3.31)	10.50 (4.00)	11.70 (3.13)	11.40 (3.31)	10.60 (4.00)

^a^ Assessed at 4 waves of measurements in childhood and adolescence (2723 participants included).

^b^ Denoting the highest level of education achieved up to 2018 (2812 participants included).

^c^ Meeting the definition for the outcome in 2006 to 2018.

### Long-term Unemployment

Overall, childhood or adolescent mental health problems were associated with long-term unemployment (Table 2). The only exception was in the comparison between persistent and episodic cases with internalizing problems (HR, 1.12; 95% CI, 0.88-1.43). The associations with the largest estimates were observed for externalizing problems: persistent cases showed increased risk of unemployment both when compared with noncases (HR, 1.87; 95% CI, 1.55-2.26) and episodic cases (HR, 1.46; 95% CI, 1.17-1.81). Most associations remained roughly unchanged after covariate adjustment (eTable 1 in Supplement 1).

**Table 2.** Hazard Ratios (HR) of Mental Health Problems in Childhood and Adolescence for Subsequent Long-term Unemployment During Follow-up

Mental health issue	Whole cohort			Exposure-discordant twins		
	No.	HR (95% CI)	*P* value	No. pairs	HR (95% CI)	*P* value
**Internalizing problems**						
Episodic vs none	2690	1.39 (1.16-1.67)	<.001	254	1.35 (0.91-2.00)	.13
Persistent vs none		1.56 (1.27-1.92)	<.001		0.96 (0.57-1.62)	.88
Persistent vs episodic		1.12 (0.88-1.43)	.37		0.71 (0.43-1.18)	.19
**Externalizing problems**						
Episodic vs none	2690	1.28 (1.08-1.53)	.005	273	1.06 (0.74-1.51)	.75
Persistent vs none		1.87 (1.55-2.26)	<.001		0.99 (0.62-1.60)	>.99
Persistent vs episodic		1.46 (1.17-1.81)	<.001		0.94 (0.58-1.54)	.81
**Internalizing and/or externalizing problems**						
Episodic vs none	2690	1.26 (1.06-1.49)	.007	344	1.00 (0.71-1.42)	>.99
Persistent vs none		1.74 (1.47-2.06)	<.001		1.00 (0.65-1.54)	>.99
Persistent vs episodic		1.38 (1.14-1.68)	.001		1.00 (0.65-1.54)	>.99

For externalizing problems, early onset was associated with increased risk of unemployment compared with later onset (HR, 1.41; 95% CI, 1.01-1.96). There was no statistically significant difference between early-onset and later-onset cases for other exposures (Table 3).

**Table 3.** Hazard Ratios (HR) for Time-of-Onset of Mental Health Problems for Subsequent Long-term Unemployment, Comparing Early-Onset With Later-Onset Cases

Mental health issue	Whole cohort^a^			Exposure-discordant twins^a^		
	No.	HR (95% CI)	*P* value	No. pairs	HR (95% CI)	*P* value
**Internalizing problems**						
Later-onset	148	1 [Reference]	.14	21	1 [Reference]	.12
Early-onset	266	0.77 (0.55-1.09)			0.40 (0.13-1.28)	
**Externalizing problems**						
Later-onset	173	1 [Reference]	.04	17	1 [Reference]	.57
Early-onset	240	1.41 (1.01-1.96)			1.40 (0.44-4.41)	
**Internalizing and/or externalizing problems**						
Later-onset	225	1 [Reference]	.66	33	1 [Reference]	.51
Early-onset	392	0.94 (0.71-1.24)			0.75 (0.32-1.78)	

^a^ Including complete cases (ie, participants assessed at all 4 waves of measurements).

HRs for the association between childhood or adolescent mental health problems and unemployment were greatly attenuated and were not statistically significant in co-twin control analysis when contrasted with the individual-level analysis (Table 2). However, for internalizing problems, an exception was observed when comparing episodic cases with noncases, with point estimates being nearly the same in both analyses, albeit not statistically significant in co-twin control analysis (HR, 1.35; 95% CI, 0.91-2.00).

### Work Disability

Childhood or adolescent mental health problems were associated with work disability (Table 4). Especially persistent cases showed higher risk of work disability than noncases: this was observed for internalizing (HR, 2.32; 95% CI, 1.80-2.99), externalizing (HR, 2.38; 95% CI, 1.87-3.03), and internalizing and/or externalizing (HR, 2.41; 95% CI, 1.95-2.99) problems. Most HRs for the association between childhood or adolescent mental health problems and work disability remained largely unchanged in co-twin control analysis when contrasted with the individual-level analysis. Most associations remained significant after covariate adjustment (eTable 2 in Supplement 1).

**Table 4.** Hazard Ratios (HR) of Mental Health Problems in Childhood and Adolescence for Subsequent Work Disability During Follow-up

Mental health issue	Whole cohort			Exposure-discordant twins		
	No.	HR (95% CI)	*P* value	No. pairs	HR (95% CI)	*P* value
**Internalizing problems**						
Episodic vs none	2690	1.66 (1.31-2.10)	<.001	254	1.67 (0.94-2.97)	.08
Persistent vs none		2.32 (1.80-2.99)	<.001		1.94 (1.00-3.77)	.049
Persistent vs episodic		1.40 (1.02-1.92)	.04		1.16 (0.64-2.13)	.62
**Externalizing problems**						
Episodic vs none	2690	1.38 (1.09-1.74)	.006	273	1.12 (0.68-1.84)	.66
Persistent vs none		2.38 (1.87-3.03)	<.001		1.92 (1.04-3.56)	.04
Persistent vs episodic		1.73 (1.28-2.32)	<.001		1.72 (0.91-3.26)	.10
**Internalizing and/or externalizing problems**						
Episodic vs none	2690	1.41 (1.12-1.78)	.003	344	0.97 (0.59-1.58)	.89
Persistent vs none		2.41 (1.95-2.99)	<.001		2.27 (1.24-4.15)	.007
Persistent vs episodic		1.71 (1.32-2.21)	<.001		2.35 (1.32-4.21)	.003

As for timing, there was no statistically significant difference between early-onset and later-onset cases regarding any of the exposures (Table 5). A similar pattern of findings was observed when including continuous measures of childhood or adolescent mental health problems (eTables 3-6 in Supplement 1).

**Table 5.** Hazard Ratios (HR) for Time-of-Onset of Mental Health Problems for Subsequent Work Disability, Comparing Early-Onset With Later-Onset Cases

Mental health issue	Whole cohort^a^			Exposure-discordant twins^a^		
	No.	HR (95% CI)	*P* value	No. pairs	HR (95% CI)	*P* value
**Internalizing problems**						
Later-onset	148	1 [Reference]	.50	21	1 [Reference]	.78
Early-onset	266	1.16 (0.76-1.76)			1.17 (0.39-3.47)	
**Externalizing problems**						
Later-onset	173	1 [Reference]	.22	17	1 [Reference]	.76
Early-onset	240	1.29 (0.86-1.91)			0.83 (0.25-2.73)	
**Internalizing and/or externalizing problems**						
Later-onset	225	1 [Reference]	.13	33	1 [Reference]	.83
Early-onset	392	1.32 (0.92-1.88)			1.10 (0.47-2.59)	

^a^ Including complete cases (ie, participants assessed at all 4 waves of measurements).

## Discussion

In this long-term follow-up study of Swedish twins, we investigated associations of early-life internalizing and externalizing problems with adult long-term unemployment and work disability. We also assessed potential confounding by familial factors.

Our findings contribute to research on mental health and adverse labor market outcomes in 3 important ways. First, our results in the whole cohort analysis replicated main findings of research suggesting that youths with depression^27,28^ and conduct problems^14,31^ are at elevated risk of adverse labor market outcomes. We found that persistent cases, in particular, had increased risk of unemployment and work disability, regardless of type of problem (ie, internalizing, externalizing). Importantly, persistent cases were found to be at higher risk when compared with noncases as well as episodic cases, with the only exception being the comparison between persistent and episodic cases with internalizing problems where no difference was found.

Second, our results based on exposure-discordant twin pairs suggested familial factors may to some extent underlie the association between childhood and adolescent mental health problems and later unemployment, and perhaps more so for persistent problems. Interestingly, we found that episodic cases with internalizing problems had virtually the same point estimates in the whole cohort analysis as in the co-twin control analysis, although the latter was not statistically significant. Conversely, we found that familial factors may be of less importance for work disability, indicating that nonshared environmental factors bear relevance for these associations. These findings extend previous work on early-life psychopathology and confirm other research results suggesting familial factors play an important role for some, but not all, LMM outcomes.^23,24,25,26^

Third, we found little evidence to support that the timing of mental health problems is related to LMM. This is in line with some findings^29^ but is opposed to others^28^ reporting poorer long-term outcomes following adolescent depression compared with childhood depression. However, for externalizing problems, we observed that cases with early onset had higher risk of unemployment than those with later onset: point estimates were almost the same in the whole cohort as in exposure-discordant twin pairs, although the latter was not statistically significant.

Understanding the etiologic (and potentially causal) mechanisms underlying the associations between childhood and adolescent mental health problems and adult LMM outcomes may help inform further research into the specific factors that increase the risk for long-term adversities. The potential effects of nonshared environmental factors, which usually are the circumstances unique to the individual (eg, life events), emphasizes the possibilities to mitigate problems early on. This may involve targeting individual or contextual risk factors such as childhood adversities.^44,45,46^ The role of nonshared environmental factors also seems promising for the development of early prevention targets that may, in effect, promote increased labor market participation in young individuals with mental health problems.^47,48^ However, further research is warranted to assess if, how, and when such targeted efforts need to be tailored for vulnerable groups.

Our findings suggest persistent psychopathology per se may be an early risk marker of work disability, regardless of type of broad-band symptomatology. Persistent cases in our combined conceptualization of internalizing and/or externalizing problems showed the highest risk of work disability. This signals the relevance of considering persistent psychopathology more broadly, consistent with emerging epidemiologic evidence on the role of a general psychopathology factor.^49^

### Strengths and Limitations

Strengths of this study included the combination of extensive survey- and registry-based data. The design of TCHAD, featuring a 4-wave data collection, enabled us to track internalizing and externalizing problems across important developmental periods in a well-characterized cohort. We included outcomes ascertained from high-quality nationwide registries, covering virtually all individuals in Sweden.^35,40,50^ This should be contrasted with the many studies relying solely on self-reported outcomes,^12^ which are prone to potential biases.^51,52^ Overall, our findings should apply to the general population of young adults, as Swedish twins are representative of the general population.^53,54^ However, our findings may not be generalizable to other countries or age cohorts.

This study also had several limitations. We relied on classification of internalizing and externalizing problems based on parent- and self-reported information. Although the CBCL captures many of the symptoms pertaining to a wide range of mental health disorders, the instrument is not recommended for diagnostic ascertainment. This may call the validity of investigated exposures into question. Moreover, we had limited possibilities to test any potentially indirect or mediating role of adult mental health problems, which precluded us from drawing conclusions about intermediate pathways involved. Furthermore, the estimates in some co-twin control analyses should be interpreted with caution given the limited number of exposure-discordant twin pairs. Finally, we were not able to assess the relative contributions from genetic vs shared environmental factors, as there were too few exposure-discordant twin pairs for further analyses stratified by zygosity.

## Conclusions

In this cohort study of young twins prospectively studied from childhood to adulthood, familial factors explained the association of early-life persistent mental health problems with future unemployment, whereas such factors seemed to be of less importance for work disability. This suggests nonshared environmental factors may play an important role for the risk of future work disability, thereby underscoring the opportunity for public mental health promotion already at young ages.

## Notes

Supplement 1. **eTable 1.** Associations Between Childhood/Adolescent Mental Health Problems and Adulthood Long-term Unemployment, With Covariate Adjustment **eTable 2.** Associations Between Childhood/Adolescent Mental Health Problems and Adulthood Work Disability, With Covariate Adjustment **eTable 3.** Associations Between Childhood/Adolescent Mental Health Problems and Adulthood Long-term Unemployment, With Continuous Exposures **eTable 4.** Associations Between Childhood/Adolescent Mental Health Problems and Adulthood Long-term Unemployment, With Continuous Exposures and Covariate Adjustment **eTable 5.** Associations Between Childhood/Adolescent Mental Health Problems and Adulthood Work Disability, With Continuous Exposures **eTable 6.** Associations Between Childhood/Adolescent Mental Health Problems and Adulthood Work Disability, With Continuous Exposures and Covariate Adjustment

Supplement 2.
Data Sharing Statement
