# Supplementary material for: Associations of Internalizing and Externalizing Problems in Childhood and Adolescence With Adult Labor Market Marginalization
Source: JAMA Netw Open. 2023 Jun 8;6(6):e2317905. doi: 10.1001/jamanetworkopen.2023.17905 (PMC10251215; doi:10.1001/jamanetworkopen.2023.17905)
Supplement: Supplement 1. — eTable 1. Associations Between Childhood/Adolescent Mental Health Problems and Adulthood Long-term Unemployment, With Covariate Adjustment eTable 2. Associations Between Childhood/Adolescent Mental Health Problems and Adulthood Work Disability, With Covariate Adjustment eTable 3. Associations Between Childhood/Adolescent Mental Health Problems and Adulthood Long-term Unemployment, With Continuous Exposures eTable 4. Associations Between Childhood/Adolescent Mental Health Problems and Adulthood Long-term Unemployment, With Continuous Exposures and Covariate Adjustment eTable 5. Associations Between Childhood/Adolescent Mental Health Problems and Adulthood Work Disability, With Continuous Exposures eTable 6. Associations Between Childhood/Adolescent Mental Health Problems and Adulthood Work Disability, With Continuous Exposures and Covariate Adjustment [file jamanetwopen-e2317905-s001.pdf]

Supplemental Online Content

Alaie I, Svedberg P, Ropponen A, Narusyte J. Associations of internalizing and externalizing problems in childhood and adolescence with adult labor market marginalization. *JAMA Netw Open.* 2023;6(6):e2317905. doi:10.1001/jamanetworkopen.2023.17905

eTable 1. Associations Between Childhood/Adolescent Mental Health Problems and Adulthood Long-term Unemployment, With Covariate Adjustment

eTable 2. Associations Between Childhood/Adolescent Mental Health Problems and Adulthood Work Disability, With Covariate Adjustment

eTable 3. Associations Between Childhood/Adolescent Mental Health Problems and Adulthood Long-term Unemployment, With Continuous Exposures

eTable 4. Associations Between Childhood/Adolescent Mental Health Problems and Adulthood Long-term Unemployment, With Continuous Exposures and Covariate Adjustment

eTable 5. Associations Between Childhood/Adolescent Mental Health Problems and Adulthood Work Disability, With Continuous Exposures

eTable 6. Associations Between Childhood/Adolescent Mental Health Problems and Adulthood Work Disability, With Continuous Exposures and Covariate Adjustment

This supplemental material has been provided by the authors to give readers additional information about their work.

© 2023 Alaie I et al. *JAMA Network Open.*

eTable 1. Associations Between Childhood/Adolescent Mental Health Problems and Adulthood Long-Term Unemployment, With Covariate Adjustment

	Model for whole cohort[a]			Model for exposure-discordant twins[b]		
	n	**HR (95% CI)**	***p***	***n pairs***	**HR (95% CI)**	***p***
Internalizing problems						
Episodic vs. none		1.33 (1.11-1.60)	0.002		1.34 (0.90-2.00)	0.147
Persistent vs. none	2679	1.48 (1.20-1.84)	<0.001	251	0.98 (0.57-1.67)	0.934
Persistent vs. episodic		1.11 (0.87-1.43)	0.394		0.73 (0.44-1.22)	0.228
Externalizing problems						
Episodic vs. none		1.15 (0.97-1.38)	0.112		1.06 (0.74-1.52)	0.734
Persistent vs. none	2679	1.56 (1.29-1.89)	<0.001	271	1.01 (0.62-1.64)	0.969
Persistent vs. episodic		1.35 (1.09-1.68)	0.007		0.95 (0.58-1.56)	0.836
Internalizing/Externalizing problems						
Episodic vs. none		1.16 (0.97-1.37)	0.096		0.98 (0.68-1.39)	0.897
Persistent vs. none	2679	1.53 (1.29-1.82)	<0.001	341	0.97 (0.61-1.52)	0.881
Persistent vs. episodic		1.32 (1.09-1.61)	0.004		0.99 (0.63-1.55)	0.960

a: Adjusted for sex and educational level
b: Adjusted for educational level

© 2023 Alaie I et al. *JAMA Network Open.*

eTable 2. Associations Between Childhood/Adolescent Mental Health Problems and Adulthood Work Disability, With Covariate Adjustment

	**Model for whole cohort[a]**			**Model for exposure-discordant twins[b]**		
	n	**HR (95% CI)**	***p***	***n pairs***	**HR (95% CI)**	***p***
Internalizing problems						
Episodic vs. none		1.54 (1.22-1.95)	<0.001		1.66 (0.91-3.02)	0.099
Persistent vs. none	2679	2.03 (1.56-2.64)	<0.001	251	1.87 (0.95-3.68)	0.068
Persistent vs. episodic		1.32 (0.96-1.82)	0.092		1.13 (0.61-2.10)	0.697
Externalizing problems						
Episodic vs. none		1.26 (1.00-1.60)	0.048		1.12 (0.68-1.86)	0.661
Persistent vs. none	2679	2.07 (1.62-2.65)	<0.001	271	1.92 (1.03-3.58)	0.040
Persistent vs. episodic		1.64 (1.22-2.20)	0.001		1.71 (0.90-3.26)	0.100
Internalizing/Externalizing problems						
Episodic vs. none		1.32 (1.05-1.66)	0.018		0.94 (0.56-1.56)	0.798
Persistent vs. none	2679	2.08 (1.67-2.59)	<0.001	341	2.17 (1.18-4.00)	0.013
Persistent vs. episodic		1.58 (1.22-2.04)	<0.001		2.32 (1.29-4.18)	0.005

a: Adjusted for sex and educational level
b: Adjusted for educational level

© 2023 Alaie I et al. *JAMA Network Open.*

eTable 3. Associations Between Childhood/Adolescent Mental Health Problems and Adulthood Long-Term Unemployment, With Continuous Exposures

	Model 1[a]			Model 2[b]		
	n	**HR (95% CI)**	***p***	***n***	**HR (95% CI)**	***p***
Internalizing problems	2690	1.031 (1.024-1.039)	<0.001	1782	1.010 (0.983-1.037)	0.469
Externalizing problems	2690	1.033 (1.026-1.040)	<0.001	1782	1.006 (0.983-1.030)	0.607
Internalizing/Externalizing problems	2690	1.043 (1.035-1.052)	<0.001	1782	1.012 (0.981-1.045)	0.439

a: Cox proportional hazards with clustered robust standard errors in the whole cohort
b: Conditional Cox proportional hazards in monozygotic and same-sexed dizygotic twin pairs

© 2023 Alaie I et al. *JAMA Network Open.*

eTable 4. Associations Between Childhood/Adolescent Mental Health Problems and Adulthood Long-Term Unemployment, With Continuous Exposures and Covariate Adjustment

	Model 1[a]			Model 2[b]		
	n	**HR (95% CI)**	***p***	***n***	**HR (95% CI)**	***p***
Internalizing problems	2679	1.027 (1.019-1.036)	<0.001	1774	1.012 (0.985-1.040)	0.382
Externalizing problems	2679	1.022 (1.014-1.029)	<0.001	1774	1.006 (0.982-1.030)	0.618
Internalizing/Externalizing problems	2679	1.032 (1.023-1.041)	<0.001	1774	1.014 (0.982-1.047)	0.392

a: Cox proportional hazards with clustered robust standard errors in the whole cohort, adjusted for sex and educational level
b: Conditional Cox proportional hazards in monozygotic and same-sexed dizygotic twin pairs, adjusted for educational level

© 2023 Alaie I et al. *JAMA Network Open.*

eTable 5. Associations Between Childhood/Adolescent Mental Health Problems and Adulthood Work Disability, With Continuous Exposures

	Model 1[a]			Model 2[b]		
	n	**HR (95% CI)**	***p***	***n***	**HR (95% CI)**	***p***
Internalizing problems	2690	1.046 (1.035-1.056)	<0.001	1782	1.036 (1.003-1.071)	0.034
Externalizing problems	2690	1.038 (1.028-1.048)	<0.001	1782	1.018 (0.988-1.049)	0.244
Internalizing/Externalizing problems	2690	1.055 (1.043-1.067)	<0.001	1782	1.042 (1.002-1.084)	0.040

a: Cox proportional hazards with clustered robust standard errors in the whole cohort
b: Conditional Cox proportional hazards in monozygotic and same-sexed dizygotic twin pairs

© 2023 Alaie I et al. *JAMA Network Open.*

eTable 6. Associations Between Childhood/Adolescent Mental Health Problems and Adulthood Work Disability, With Continuous Exposures and Covariate Adjustment

	Model 1[a]			Model 2[b]		
	n	**HR (95% CI)**	***p***	***n***	**HR (95% CI)**	***p***
Internalizing problems	2679	1.038 (1.028-1.048)	<0.001	1774	1.031 (0.997-1.067)	0.072
Externalizing problems	2679	1.030 (1.021-1.040)	<0.001	1774	1.016 (0.985-1.047)	0.325
Internalizing/Externalizing problems	2679	1.044 (1.033-1.056)	<0.001	1774	1.036 (0.996-1.079)	0.081

a: Cox proportional hazards with clustered robust standard errors in the whole cohort, adjusted for sex and educational level
b: Conditional Cox proportional hazards in monozygotic and same-sexed dizygotic twin pairs, adjusted for educational level

© 2023 Alaie I et al. *JAMA Network Open.*
