# Supplementary material for: Associations of Internalizing and Externalizing Problems in Childhood and Adolescence With Adult Labor Market Marginalization
Source: JAMA Netw Open. 2023 Jun 8;6(6):e2317905. doi: 10.1001/jamanetworkopen.2023.17905 (PMC10251215; doi:10.1001/jamanetworkopen.2023.17905)
Supplement: Supplement 2. — Data Sharing Statement [file jamanetwopen-e2317905-s002.pdf]

Data Sharing Statement

Alaie. Associations of Internalizing and Externalizing Problems in Childhood and Adolescence With Adult Labor Market Marginalization. *JAMA Netw Open*. Published June 08, 2023. doi:10.1001/jamanetworkopen.2023.17905

Data
Data available: No

Additional Information
Explanation for why data not available: The data that support the findings of this study are available from the original sources: the Swedish Twin Registry, Statistics Sweden, Swedish Social Insurance Agency, and the National Board of Health and Welfare. Restrictions apply to the availability of the data used in this study based on the Swedish Twin project Of Disability pension and Sickness absence (STODS), which were used with ethical permission for the current study and therefore are not publicly available. According to the General Data Protection Regulation, the Swedish law SFS 2018:218, the Swedish Data Protection Act, the Swedish Ethical Review Act, and the Public Access to Information and Secrecy Act, this type of sensitive data can be made available only after legal review, for researchers who meet the criteria for access to this type of sensitive and confidential data. Readers may contact the last author regarding the details.
